# High-Resolution Cryo-Electron Microscopy Structure Determination of *Haemophilus influenzae* Tellurite-Resistance Protein A via 200 kV Transmission Electron Microscopy

**DOI:** 10.3390/ijms25084528

**Published:** 2024-04-20

**Authors:** Nhi L. Tran, Skerdi Senko, Kyle W. Lucier, Ashlyn C. Farwell, Sabrina M. Silva, Phat V. Dip, Nicole Poweleit, Giovanna Scapin, Claudio Catalano

**Affiliations:** NanoImaging Services, 4940 Carroll Canyon Road, Suite 115, San Diego, CA 92121, USA; ntran@nanoimagingservices.com (N.L.T.); klucier@nanoimagingservices.com (K.W.L.); afarwell@nanoimagingservices.com (A.C.F.);

**Keywords:** cryo-EM, 200 kV, membrane protein, fully embed, alpha-helical, detergents, structural biology

## Abstract

Membrane proteins constitute about 20% of the human proteome and play crucial roles in cellular functions. However, a complete understanding of their structure and function is limited by their hydrophobic nature, which poses significant challenges in purification and stabilization. Detergents, essential in the isolation process, risk destabilizing or altering the proteins’ native conformations, thus affecting stability and functionality. This study leverages single-particle cryo-electron microscopy to elucidate the structural nuances of membrane proteins, focusing on the SLAC1 bacterial homolog from *Haemophilus influenzae* (*Hi*TehA) purified with diverse detergents, including n-dodecyl β-D-maltopyranoside (DDM), glycodiosgenin (GDN), β-D-octyl-glucoside (OG), and lauryl maltose neopentyl glycol (LMNG). This research not only contributes to the understanding of membrane protein structures but also addresses detergent effects on protein purification. By showcasing that the overall structural integrity of the channel is preserved, our study underscores the intricate interplay between proteins and detergents, offering insightful implications for drug design and membrane biology.

## 1. Introduction

Membrane proteins (MPs) are integral components of cellular machinery, orchestrating a myriad of critical functions such as signal transduction, energy metabolism, and molecular transport. These proteins, which constitute about 20% of the human proteome, are of paramount importance in pharmaceutical research, particularly the G-protein coupled receptor (GPCR) superfamily, known for being a prime target in drug discovery efforts [1,2]. Understanding the intricate molecular architecture of MPs is fundamental to deciphering their functionality, where a detailed analysis of their atomic arrangement can shed light on their biochemical mechanisms, thus addressing vital biological queries and facilitating the development of therapeutic interventions.

Nevertheless, the path to elucidating the structural complexities of MPs is fraught with challenges, primarily due to their intrinsic hydrophobic nature, which complicates their purification and stabilization [3]. Detergents, with their amphipathic properties, have traditionally been the cornerstone in the isolation and purification processes of MPs, playing a critical role in the initial stages of protein reconstitution. These molecules, characterized by their hydrophilic head groups and hydrophobic alkyl tails, operate at the juncture of free monomers and micelles, governed by the critical micelle concentration (CMC), which dictates the threshold for stable micelle formation [4,5]. Despite their utility, the application of detergents in MP purification is a double-edged sword, presenting obstacles that impede the characterization of protein structures due to their potential to destabilize or alter the protein’s native conformation [6,7,8]. The specificity of protein-detergent interactions necessitates meticulous selection of the appropriate detergent, a task that is both critical and challenging for preserving the protein’s stability and functionality [6,7,8]. This selection process is complicated further by the potential of detergents to induce structural deviations and interfere with subsequent biophysical analyses through the introduction of background noise or by affecting experimental conditions [9,10].

Despite these challenges, significant strides have been made in the field of structural biology, as evidenced by the deposition of numerous membrane protein structures in the Protein Data Bank [11,12]. In an attempt to understand the role that different detergents may play in an MP structure, we analyzed single-particle cryo-electron microscopy (cryo-EM) structures of the SLAC1 bacterial homolog from *Haemophilus influenzae* (*Hi*TehA), purified using n-dodecyl β-D-maltopyranoside (DDM), glycodiosgenin (GDN), β-D-octyl-glucoside (OG), and lauryl maltose neopentyl glycol (LMNG). The SLAC1 homolog, *Hi*TehA, although not completely biochemically characterized, is identified as a transporter protein involved in tellurite resistance and adaptation to environmental stresses, highlighting its role in bacterial survival through regulating solute concentrations [13]. TehA is a relatively small protein (a trimer of 37 kDa monomers), almost completely encapsulated within the detergent micelle. Imaging these kinds of samples using cryo-EM presents significant challenges because of the presence of the micelle which hinders the proper alignment of projections necessary for the reconstruction process. A helpful strategy is to use binding proteins, which significantly aid in the reconstruction process by enhancing the mass and contrast of the MP, serving as molecular markers that help overcome the constraints of existing algorithms [14,15]. The obstacles traditionally associated with cryo-EM are progressively diminishing owing to advancements in both experimental and computational methodologies, as demonstrated by the work of Motiwala et al. [16] as well as the work of other groups [17,18,19,20]. This progress has inspired us to pursue the reconstruction of *Hi*TehA without the assistance of a binding protein and utilizing a standard 200 kV microscope (ThermoFischer Scientific, Waltham, MA, USA) and workflows. This research enhances our understanding of membrane protein architecture while also examining potential effects of detergents on protein structure, thus advancing membrane protein research.

## 2. Results

### 2.1. Structure Determination of HiTehA

To investigate the impact of selected detergents on the structural properties of *Hi*TehA, we employed cryo-EM for high-resolution structure determination. We successfully elucidated the structures of all four samples with an average resolution of 3 Å. The *Hi*TehA purified using GDN achieved the highest resolution of 2.9 Å, followed by those purified with DDM and LMNG at resolutions of 3.1 Å, and OG at 3.2 Å, as detailed in Table 1 and Figure S1.

**Figure 1:**
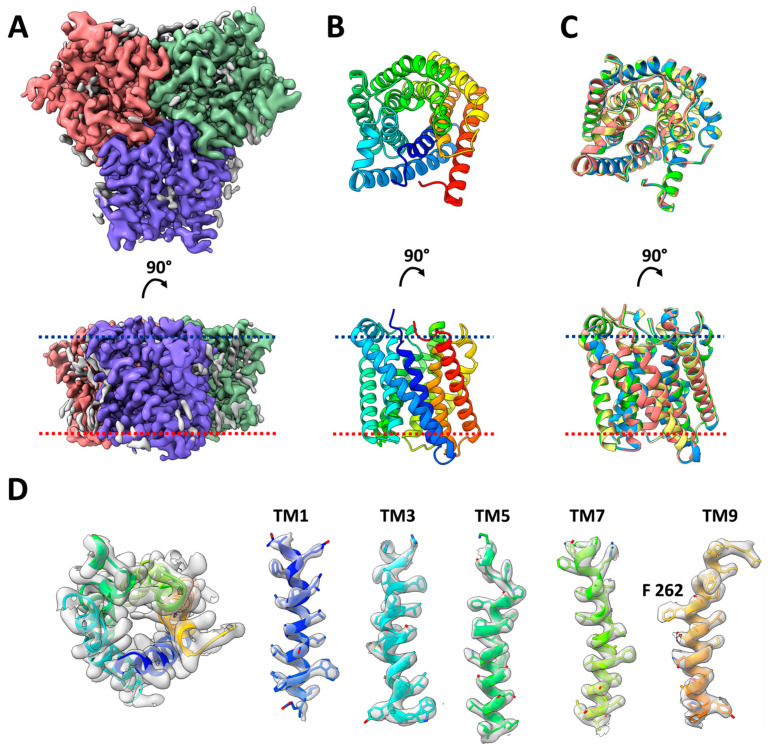
Cryo-EM structure of *HiTehA*. (**A**) Cryo-EM density map of *HiTehA* trimer purified in β-D-octyl-glucoside, top view (**top**) and side view (**bottom**). The monomers are distinctly colored in purple, green, and magenta, while the grey surface signifies non-standard residues such as lipids and detergent molecules. (**B**) A cartoon representation of *HiTehA* is shown from the extracellular region (**top**) and in parallel to the membrane (**bottom**). The structure is color-coded in a rainbow gradient, ranging from TM1 in blue to TM10 in red. The dotted red line indicates the extracellular side of the membrane, while the blue line represents the cytoplasmic side. (**C**) A comparison of four distinct structures for *HiTehA* purified with different detergents: DDM (magenta), LMNG (cyan), GDN (green), and OG (yellow). The views encompass both the extracellular region (**top**) and parallel to the membrane (**bottom**), highlighting the absence of structural variations influenced by the choice of detergent (**D**) Pore-lining residues in the *Hi*TehA purified with OG (**left**). Cartoon structure colored as in (**B**) fitted into the cryo-EM density map (grey). The key aspects include a clear presentation of the rolled-open structure, with the pore-lining residues depicted on the TModd helices. Notably, the gate-keeping residue Phe262 is highlighted, showcasing a clear density that emphasizes its crucial role in regulating the channel.

The cryo-EM map, encompassing the channel pore and the loops connecting each α-helix, consistently displayed well-resolved features (Figure 1A). This high level of clarity facilitated the unambiguous assignment of the protein structure, including the individual amino-acid side chains (Figure S2). *Hi*TehA is a trimeric protein; each monomer comprises 10 transmembrane helices (TMs) connected by short extracellular loops and slightly longer intracellular loops; a short helix is part of the loop connecting TM2. *Hi*TehA has no significant extracellular or intracellular domains to act as fiducial markers in determining membrane orientation. The ion channel architecture features a distinctive organization: the pore-forming helices are positioned at odd-numbered TMs, while the even TMs collectively form an outer shell (Figure 1B). This outer shell contributes significantly to the overall stability and functionality of the channel, potentially playing a pivotal role in sensing environmental changes, responding to stimuli, and regulating the opening and closing dynamics of the channels.

The four structures of *Hi*TehA closely align, revealing a minimal root-mean-square deviation (r.m.s.d.) of 0.13 Å for all atoms, except for the sample purified in DDM, which showed a slightly higher deviation of 0.26 Å (Figure 1C). This discrepancy may be attributed to distinct folding patterns in the intracellular loops between TM6 and TM7.

In general, the structural integrity of the trimeric arrangement remains unaffected using four different detergents during protein purification. Remarkably, none of the detergents tested appear to induce any significant alteration in the position of the gating Phe262 located on TM9 (Figure 1D). This observation underscores the robustness of the trimeric structure and the stability of key functional elements within HiTehA, even under varying detergent conditions.

### 2.2. Comparison of Cryo-EM and X-ray Structures

**Figure 2:**
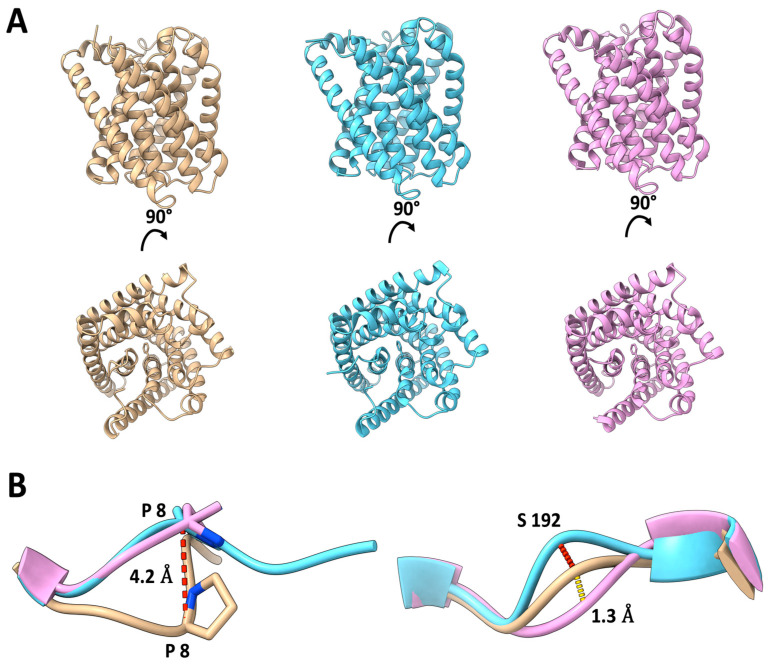
Comparative analysis of *Hi*TehA structures: Cryo-EM, cryogenic X-ray, and room temperature X-ray. (**A**) A cartoon representation of *Hi*TehA is shown from the extracellular region (**top**) and in parallel to the membrane (**bottom**) with Phe262 highlighted in sticks. The structures are color-coded in gold (cryo-EM), cyan (cryogenic X-ray) and magenta (room temperature X-ray). (**B**) Two discernible shifts are observed in the N-terminus (**left**) and the loop connecting TM6 and TM7 (**right**). Cartoon structure colored as in (**A**).

In our analysis, presented in Figure 2, we offer a detailed comparative study of the cryo-EM structures obtained from our research alongside previously established structural data [13,21]. Since both X-ray structures described below were obtained in the presence of n-octyl-β-D-glucoside (OG), we used the cryo-EM structure of HiTehA, determined using n-octyl-β-D-glucoside (OG) as detergent, which yielded a resolution of 3.1 Å. The two X-ray structures used are the 1.2 Å structure of HiTehA obtained under cryogenic conditions, which we use as a benchmark for structural clarity and precision [13], and the room temperature X-ray structure of HiTehA, resolved at 2.30 Å [21]. Overall, our cryo-EM structure exhibits a high degree of similarity with the two crystal structures, as evidenced by r.m.s.d values of 0.4 Å (PDB ID: 3M71) and 0.37 Å (PDB ID: 4YCR), respectively, highlighting the accuracy and reliability of our structural determination.

A more in-depth analysis of the structures reveals subtle yet significant shifts, particularly in the N-terminus and the loop bridging transmembrane helices 6 and 7 (TM6 and TM7). These structural shifts were quantified with maximum distances of 4.2 Å at the Cα atom of proline 8 (Pro8) and 1.3 Å at the Cα atom of serine 192 (Ser192). The N-terminal region is near the crystallographic interface (not the trimer interface), and the loop containing Ser192 has at least two conformations in the cryogenic structure. The cryo-EM analysis may be able to better capture in one experiment flexibility that is present in the structure. In any case, these variations do not structurally affect the critical gating residue Phe262 located on TM9. Given the high structural conservation observed among the different samples, we would not expect the small changes described above to impede the channel’s functionality. This observation underscores the structural resilience of *Hi*TehA, maintaining its functional architecture despite minor positional deviations.

Similarly to the findings of Axford et al. (2015), our cryo-EM map identifies a distinct density within the channel cavity on its cytoplasmic side [21]. This density is most pronounced in the structure of OG detergent, consistent with the density that was attributed to an OG detergent molecule penetrating deeply into the channel in Axford et al. The detergent has hydrophobic interactions with key residues including Phe262, Ile203, Leu18, Leu144, Leu85, and Phe82. This interaction scenario suggests that the detergent lipid polar head aligns near polar amino acids, such as Arg97 and Gln196, suggesting a complex interplay between the channel’s structural components and the detergent molecule. A more thorough examination across all four cryo-EM maps, involving a rigid fitting of the room-temperature crystal structure (PDB ID: 4YCR) on each map, revealed a linear density across all detergent conditions (as illustrated in Figure S3). However, the density was more pronounced and well resolved in the OG condition than for the other three detergents. The fact that the density is present in all four structures suggests that the observed feature is not exclusive to the use of OG detergent but could represent some other hydrophobic molecule captured by the channel during purification.

## 3. Discussion

In our study, utilizing single-particle cryo-electron microscopy (cryo-EM), we aimed to provide elucidation of the possible differences in structural details of *Hi*TehA purified with different detergents. Using a 200 kV accelerating voltage microscope, we solved the four structures reported here to near-atomic resolution, thus allowing for meaningful comparisons. This research highlights the stability and functional integrity of the trimeric *Hi*TehA across various detergent environments, with critical functional elements, such as the gating Phe262 on TM9, remaining unaltered. It also shows that the cryo-EM structure determination of membrane proteins that are fully embedded in the micelle and lack clear fiducial markers is possible, even with a 200 kV microscope. A side-by-side comparison with existing crystallographic structures supports the accuracy of our cryo-EM-derived structures. Despite observable shifts in certain regions, the overall structural integrity of the channel is preserved, and different detergents do not apparently affect the stability of the channel. The identification of a linear density within the channel cavity across all detergent conditions suggests this cavity can be occupied either by detergent molecules or by other hydrophobic molecules such as co-purified lipids. By obtaining the structures in four different detergents, we also demonstrated how cryo-EM can overcome some of the limitations of X-ray crystallography, including the challenge of obtaining crystals that yield satisfactory diffraction, as described by Chen et al. [13].

In conclusion, our work further contributes to the structural biology landscape, particularly to membrane protein structure-function research. Although the four detergents examined did not significantly affect the structure of the MP, the importance of examining detergent effects on MP structures remains an important point. This study demonstrates that it is possible to determine structures of small, fully membrane-embedded membrane proteins using cryo-EM, thus offering new methods to structurally investigate the functional interplay between proteins, detergents, and lipids.

## 4. Materials and Methods

### 4.1. Protein Expression and Purification

**Figure 3:**
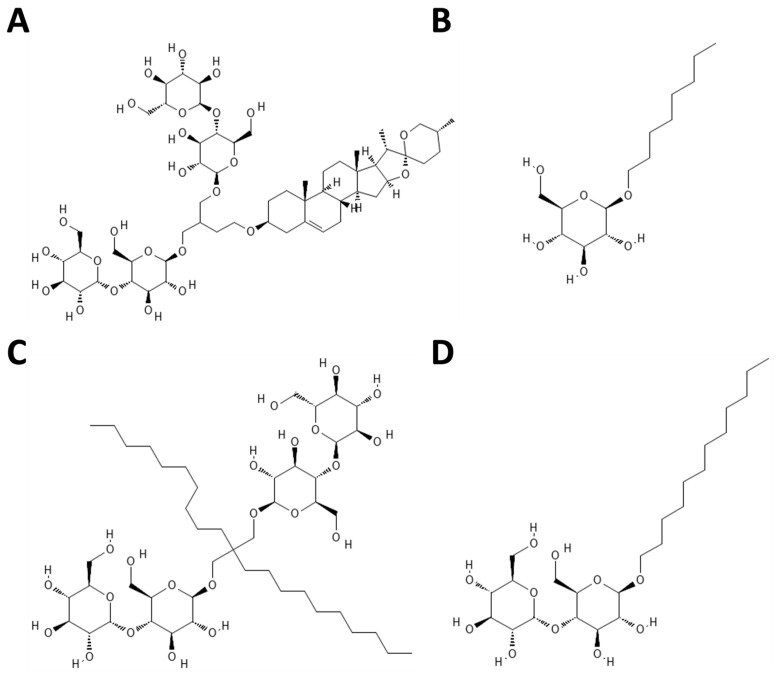
Chemical structures of the four detergents used for *Hi*TehA extraction and purification. (**A**) Glyco-diosgenin (GDN). (**B**) ß-D-glucopyranoside (OG). (**C**) lauryl maltose neopentyl glycol (LMNG). (**D**) n-dodecyl-ß-maltoside (DDM).

*Hi*TehA was expressed in *E. coli*, followed by extraction and purification in n-dodecyl-ß-maltoside (DDM) using immobilized metal affinity and size-exclusion chromatography. Subsequently, the purified TehA in DDM underwent detergent exchange into three alternative detergents: glyco-diosgenin (GDN), lauryl maltose neopentyl glycol (LMNG), and ß-D-glucopyranoside (OG) through size-exclusion chromatography (Figure 3). For a more comprehensive protocol, refer to the detailed procedure outlined by Chen et al. (2010) [13].

### 4.2. Sample Preparation

Samples were diluted to 3.0 mg/mL from their respective stocks, and 3.0 µL was applied onto glow discharge for 60 s on a PELCO easiGlow™ Glow Discharge Cleaning System (Ted Pella, Inc., Redding, CA, USA); UltrAuFoil 300 mesh holey gold UltrAuFoil R 1.2/1.3 grids; and R 1/1 grids for TehA purified in GDN (Quantifoil Micro Tools GmbH, Jena, Germany). Grid preparation was performed using a Vitrobot Mark IV (Thermo Fisher Scientific, Waltham, MA, USA), with the environmental chamber set to 95% humidity, and a temperature of 4 °C. The blotting condition parameters are described in Table 1.

Samples were imaged on a Glacios Cryo-Transmission Electron Microscope (Thermo Fisher Scientific, Waltham, MA, USA) equipped with a Falcon 4 camera (Thermo Fisher Scientific, Waltham, MA, USA) utilizing a 50 µm C2 aperture and 100 µm objective lens. Movies were acquired using the Leginon software version 3.6 at a nominal magnification of 240,000× with a calibrated pixel size of 0.566 Å and a dose rate of 10.41 e^−^/Å^2^/s with a total exposure of 3.50 s, for an accumulated dose of 36.43 e^−^/Å^2^ [22,23]. Movies were collected at a nominal defocus range of −0.5 µm to −1.5 µm.

### 4.3. Data Processing

For all datasets, data processing procedures followed highly similar strategies. TehA purified in GDN will be used as an example of the general workflow. On-the-fly data preprocessing and quality control were performed within cryoSPARC live [24]. Patch motion correction was used for correcting beam-induced motion and to account for stage drift [24]. The contrast-transfer function was estimated for each micrograph using patch CTF [25,26]. Micrographs with poor contrast-transfer function fits lower than 5 Å were removed. Then, 227,571 particles were picked with a Topaz pre-trained neural network model and extracted with a box size of 440 pixels from 4153 manually curated micrographs [27]. An initial clean-up of the extracted particles was performed by iterative rounds of 2D classifications, yielding 172,420 particles. The selected particles were used in an ab initio reconstruction followed by heterogeneous refinement. The best-resolved structure after heterogeneous refinement of 40,701 particles was used for non-uniform (NU) refinement with an imposed C3 symmetry. This map was selected for further refinement using CTF local and global refinement followed by local resolution refinement. The estimated resolution is 2.9 Å based on the gold standard Fourier shell correlation of 0.143. A full summary of the processing workflow can be found in Figure S1 and Table 1.

### 4.4. Model Building

Model building and refinement were initiated with a published model for TehA (PDB ID: 3M71). The four structures were placed into the sharpened density maps using the ChimeraX-1.2.5 fit-in map [28]. Iterative rounds of model building and refinement were performed in PHENIX v.1.19.2 and COOT v. 0.9.6 EL [29,30]. The final models were validated against the half-maps and their quality was assessed by MolProbity4.5.2 [31]. For a full summary of the data, refer to Table 1.

## Figures and Tables

**Table 1:** Summary of cryo-EM data collection, processing, and refinement statistics.

Parameter	TehA:GDN	TehA:LMNG	TehA:DDM	TehA:OG
Data Collection				
Blot Time	10 s	10 s	10 s	3 s
Blot Force	10	10	10	25
Wait Time	0 s	20 s	20 s	0 s
Microscope	Glacios	Glacios	Glacios	Glacios
Voltage (kV)	200 kV	200 kV	200 kV	200 kV
Magnification	240,000	240,000	240,000	240,000
Detector	Falcon 4	Falcon 4	Falcon 4	Falcon 4
Pixel Size (Å/pixel)	0.566	0.566	0.566	0.566
Total Electron Dose	36.43 e^−^/Å^2^	36.43 e^−^/Å^2^	36.43 e^−^/Å^2^	36.43 e^−^/Å^2^
EER Internal Frames	840	840	840	840
Defocus Range (μm)	0.5–1.5	0.5–1.5	0.5–1.5	0.5–1.5
Micrograph Images (no.)	7352	11370	3585	8354
3D Reconstruction				
Software	cryoSPARC 4.4	cryoSPARC 4.4	cryoSPARC 4.4	cryoSPARC 4.4
Final Number of Particles in the Reconstruction	40,701	36,758	63,824	30,170
Imposed Symmetry	C3	C3	C3	C3
Fourier Shell Correlation (FSC) Cut-Off	0.143	0.143	0.143	0.143
Map Resolution (Å)	2.9	3.1	3.1	3.2
Atomic Modelling and Refinement Statistics				
Software	COOT, Phenix	COOT, Phenix	COOT, Phenix	COOT, Phenix
Homology Model (PDB Accession Code)	3M71	3M71	3M71	3M71
B-factor (Å^2^)	82.1	87.7	106.1	104.7
Total Number of Atoms	7359	7359	7382	7359
Protein Residues	924	924	924	924
RMSD^2^ bond length(Å)	0.002	0.003	0.002	0.003
RMSD^2^ bond angles (°)	0.436	0.463	0.456	0.494
Molprobity Score	1.22	1.24	1.40	1.24
All Atom Clash Score	4.45	4.66	3.83	4.66
Ramachandran Plot (%)				
Favored	98.80	98.26	96.51	98.47
Allowed	1.20	1.74	3.49	1.53
Outliers	0.00	0.00	0.00	0.00
Data				
Box Pixel Size	440/440/440	440/440/440	440/440/440	440/440/440
Angles (^o^)	90.00, 90.00, 90.00	90.00, 90.00, 90.00	90.00, 90.00, 90.00	90.00, 90.00, 90.00
Resolution Estimates (Å)	Masked-Unmasked	Masked-Unmasked	Masked-Unmasked	Masked-Unmasked
d FSC (half maps; 0.143)	3.0–3.2	3.1–3.2	3.2–3.3	3.2–3.4
d 99 (full/half1/half2)	3.4/1.2/1.2–3.3/1.1/1.1	3.4/1.1/1.1–3.4/1.1/1.1	3.5/1.2/1.2–3.5/1.1/1.1	3.5/1.2/1.2–3.4/1.1/1.1
d Model	3.3–3.3	3.4–3.4	3.5–3.5	3.5–3.5
d FSC Model (0/0.143/0.5)	2.9/2.9/3.2–2.9/3.0/3.2	3.0/3.0/3.2–3.0/3.1/3.3	3.0/3.1/3.3–3.0/3.1/3.4	3.1/3.1/3.3–3.1/3.2/3.4
Map min/max/mean	−0.36/0.59/0.02	−0.34/0.58/0.02	−0.31/0.53/0.02	−0.36/0.55/0.01
Model vs. Data				
CC (Mask)	0.88	0.88	0.85	0.85
CC (Box)	0.70	0.70	0.62	0.63
CC (Peaks)	0.68	0.68	0.60	0.59
CC (Volume)	0.82	0.82	0.83	0.84

## Data Availability

EM maps and atomic models were deposited to the Electron Microscopy Data Bank (EMDB) and Protein Data Bank (PDB) databases. PDB codes for the various structures reported in this manuscript are 8VI5, 8VI4, 8VI3, and 8VI2, and the EMDB accession codes are EMD-43249, EMD-43248, EMD-43247, and EMD-43246 for TehA purified in OG, TehA purified in LMNG, TehA purified in GDN, and in TehA purified in DDM.

## Supplementary Materials

The following supporting information can be downloaded at: https://www.mdpi.com/article/10.3390/ijms25084528/s1.
