# Supplementary material for: High-Resolution Cryo-Electron Microscopy Structure Determination of Haemophilus influenzae Tellurite-Resistance Protein A via 200 kV Transmission Electron Microscopy
Source: Int J Mol Sci. 2024 Apr 20;25(8):4528. doi: 10.3390/ijms25084528 (PMC11050165; doi:10.3390/ijms25084528)
Supplement: Supplementary file 1 [file ijms-25-04528-s001.zip › ijms-2965108-supplementary.pdf]

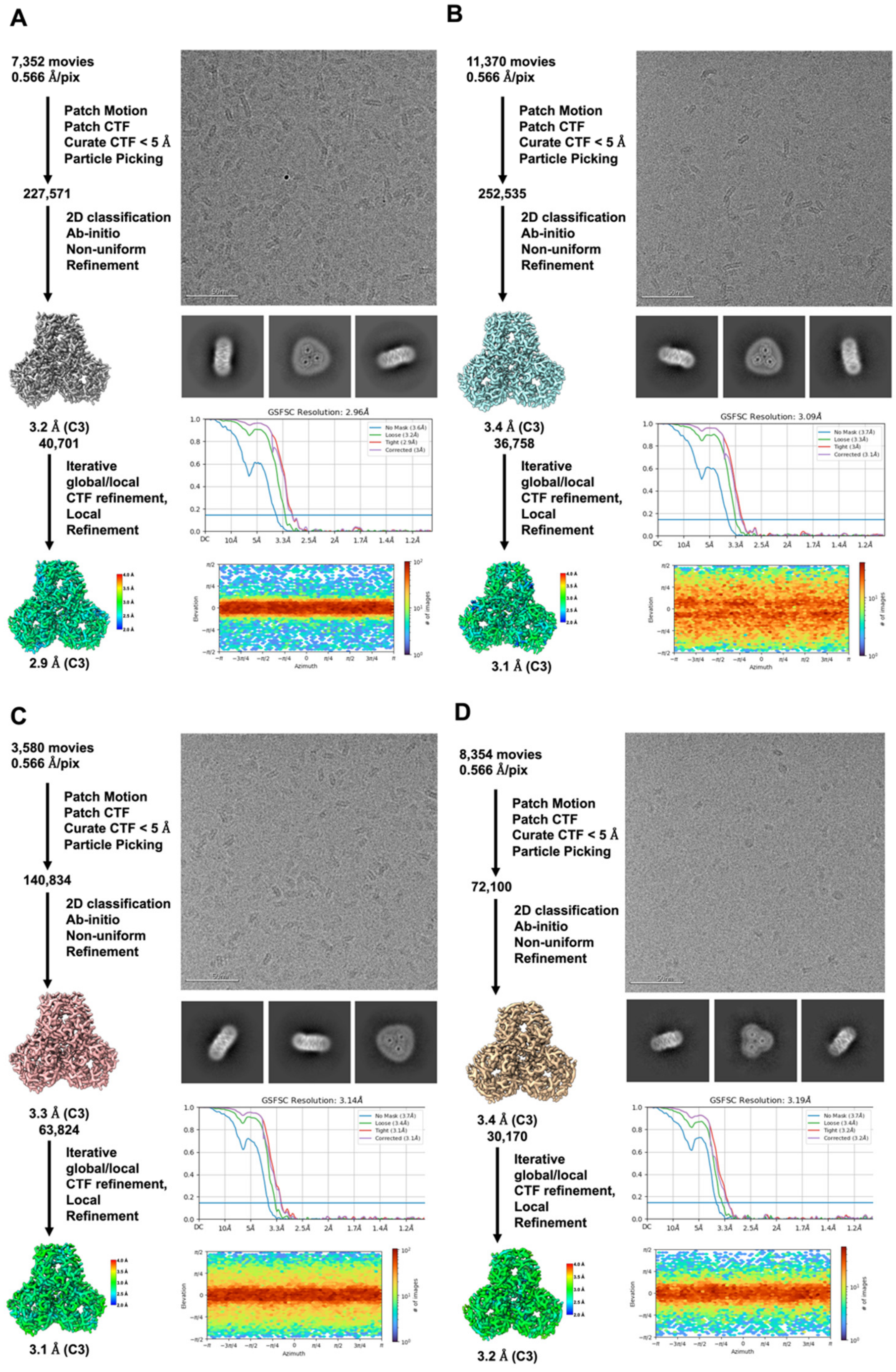

Figure S1: Summary of data processing. Processing workflow, exemplary micrograph, 2D classes, overall map, FSC curve and viewing distribution as calculated by cryoSPARC[1] for A TehA purified in GDN; B TehA purified in LMNG; C TehA purified in DDM and D TehA purified in OG.

Support Information

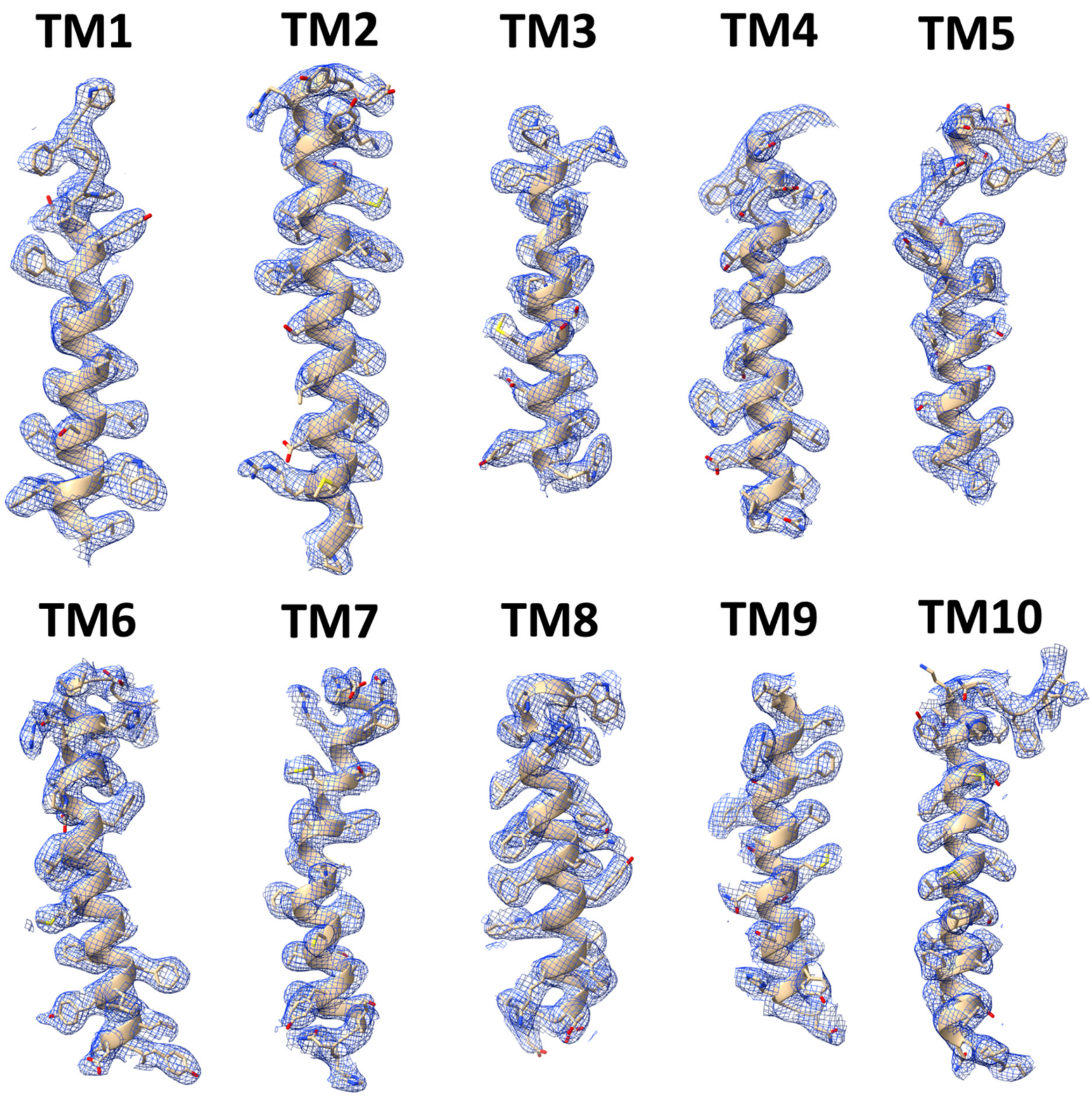

Figure S2 An example of the quality of the map for the *Hi*TehA purified in OG.

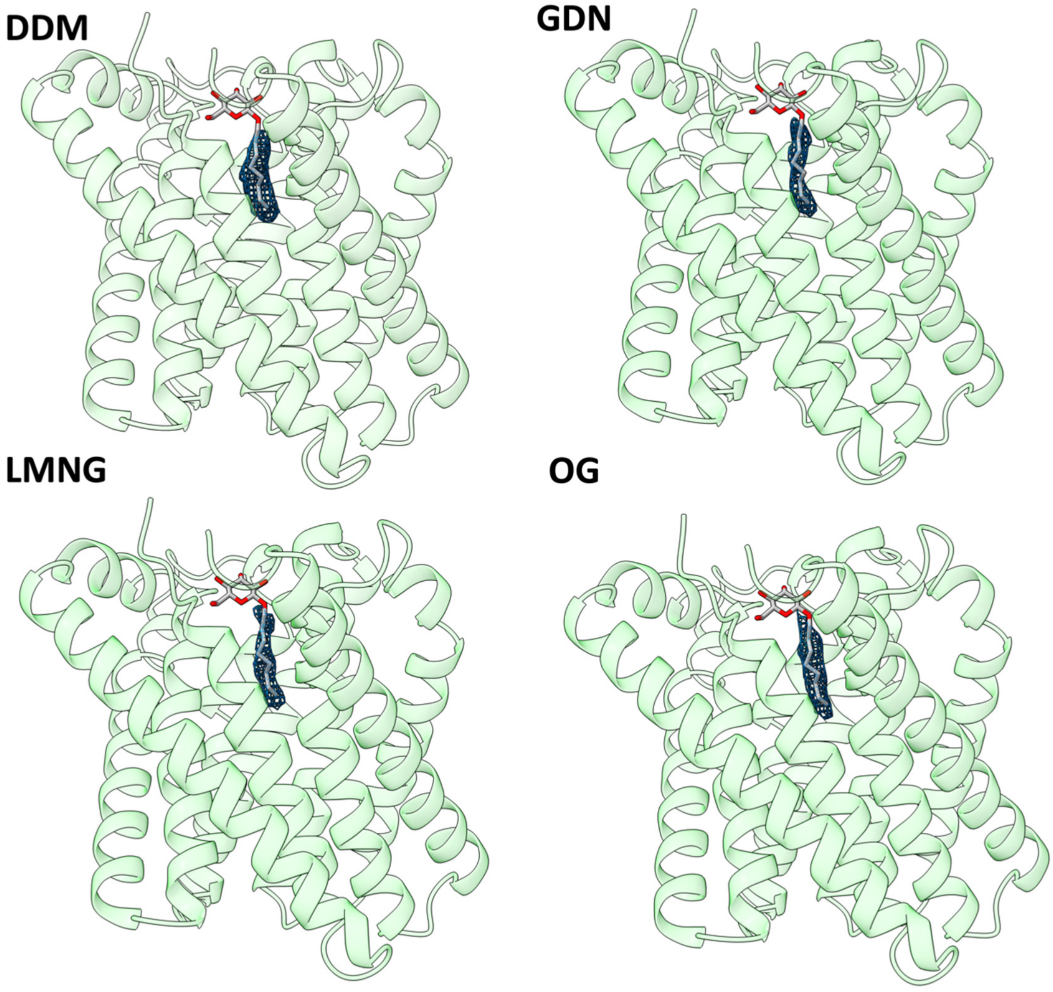

Figure S3 Comparing the cryo-EM maps of pore density with the room temperature structure rigidly fitted into the maps, transmembrane segments (TMs) are color-coded in green. Additionally, the OG molecule is represented as sticks, color-coded according to its heteroatoms, with the surrounding density map displayed as a blue mesh.

1. Punjani, A., Rubinstein, J. L., Fleet, D. J. & Brubaker, M. A. cryoSPARC: algorithms for rapid unsupervised cryo-EM structure determination. *Nat Methods* **14**, 290–296 (2017).
